# C-Reactive Protein for Pulmonary Tuberculosis Screening and Treatment Response Monitoring in Children

**DOI:** 10.1093/ofid/ofaf816

**Published:** 2026-01-07

**Authors:** Joy Githua, Jerphason Mecha, Joshua Stern, Jaclyn N Escudero, Lilian Njagi, Lucy Kijaro, Jacqueline Mirera, Wilfred Murithi, Grace John-Stewart, Elizabeth Maleche-Obimbo, Videlis Nduba, Sylvia M LaCourse

**Affiliations:** Centre for Respiratory Diseases Research, Kenya Medical Research Institute, Nairobi, Kenya; Department of Medical Microbiology and Immunology, University of Nairobi, Nairobi, Kenya; Centre for Respiratory Diseases Research, Kenya Medical Research Institute, Nairobi, Kenya; Department of Mathematics, University of Nairobi, Nairobi, Kenya; Department of Global Health, University of Washington, Seattle, Washington, USA; Department of Global Health, University of Washington, Seattle, Washington, USA; Centre for Respiratory Diseases Research, Kenya Medical Research Institute, Nairobi, Kenya; Centre for Respiratory Diseases Research, Kenya Medical Research Institute, Nairobi, Kenya; Centre for Respiratory Diseases Research, Kenya Medical Research Institute, Nairobi, Kenya; Centre for Respiratory Diseases Research, Kenya Medical Research Institute, Nairobi, Kenya; Department of Global Health, University of Washington, Seattle, Washington, USA; Department of Medicine, University of Washington, Seattle, Washington, USA; Department of Epidemiology, University of Washington, Seattle, Washington, USA; Department of Pediatrics, University of Washington, Seattle, Washington, USA; Department of Pediatrics and Child Health, University of Nairobi, Nairobi, Kenya; Department of Pediatrics, Kenyatta National Hospital, Nairobi, Kenya; Centre for Respiratory Diseases Research, Kenya Medical Research Institute, Nairobi, Kenya; Department of Global Health, University of Washington, Seattle, Washington, USA; Department of Medicine, University of Washington, Seattle, Washington, USA; Department of Epidemiology, University of Washington, Seattle, Washington, USA

**Keywords:** C-reactive protein, diagnosis, pediatric, treatment response, tuberculosis

## Abstract

C-reactive protein (CRP) was evaluated as a biomarker for pulmonary tuberculosis (TB) diagnosis and treatment response monitoring in 292 Kenyan children. Although diagnostic sensitivity was suboptimal (35.5%–50.0%), the median CRP level decreased during TB treatment in children with confirmed (*P* = .02) or unconfirmed (*P* < .001) TB, primarily among those with baseline CRP elevation ≥5 mg/L (40% [39 of 97]).

## Teaser

Baseline C-reactive protein (CRP) levels were similar in children with and without tuberculosis, but decreased significantly during treatment in confirmed (*P* = .02) and unconfirmed (*P* < .001) tuberculosis. While CRP's diagnostic screening performance was suboptimal, findings suggest potential utility for treatment response monitoring.

Most tuberculosis (TB)-related pediatric deaths occur in children <5 years old, primarily due to missed diagnoses [1]. Young children frequently present with disseminated or extrapulmonary TB, making diagnosis with conventional respiratory sampling methods challenging [2]. The limitations of current respiratory-based diagnostic methods highlight the need for non–sputum-based diagnostics for pediatric TB detection and treatment response monitoring [3].

C-reactive protein (CRP), an acute-phase protein associated with infectious and inflammatory conditions, including TB, has shown potential for screening and monitoring treatment response, mainly in adult TB [4]. The World Health Organization (WHO) currently endorses CRP for TB screening in people living with human immunodeficiency virus (HIV), though its utility in HIV-negative adults and pediatric populations remains underresearched [5, 6]. We evaluated CRP as a diagnostic screening and treatment response monitoring tool in a cohort of Kenyan children presenting with symptoms suggestive of TB.

## METHODS

Our prospective observational study enrolled children ≤15 years presenting to both inpatient wards and outpatient National TB Program and HIV care clinics in Nairobi, Kenya, with suggestive TB symptoms (including persistent cough, fever, night sweats, weight loss/poor weight gain, and lethargy) and children highly suspected of having TB or recommended for treatment. Children treated for TB for >7 days or planning to leave Nairobi County were excluded.

Participants underwent comprehensive evaluation including interviews, medical record review, physical examination, HIV testing, chest radiography, collection of respiratory specimens for testing by Xpert Ultra, microscopy, and culture. Chest radiographs were interpreted by a radiologist and study clinician with an additional review by a pediatric pulmonologist in case of discrepancies and classified as suggestive or not suggestive of TB using standardized forms. TB treatment followed national guidelines. All children regardless of TB diagnoses were seen at follow-up visits to assess for symptom resolution and anthropometric changes and treatment response for those who initiated treatment.

Participants were categorized as following using National Institutes of Health consensus definitions: confirmed TB (positive Xpert/Ultra results and/or culture), unconfirmed TB (symptoms consistent with TB plus supporting evidence but negative Xpert results and culture), or unlikely TB (symptoms present but not meeting unconfirmed TB criteria) post hoc. Participants whose symptoms resolved without initiation of TB treatment were classified as unlikely TB, and those with symptoms who did not meet criteria for unconfirmed TB and lack sufficient follow-up were categorized as “unclassifiable” [7].

Serum CRP levels were measured using a COBAS C111 chemistry analyzer (Roche Diagnostics) at baseline for all participants and during follow-up visits primarily for participants on TB treatment. The analytical sensitivity was 0.6–350 mg/L. Normal pediatric ranges were defined as <5 mg/L based on manufacturer specifications and WHO recommendations [8]. Due to an external laboratory reporting error during March–April 2023, a subset of samples (57 samples from 54 participants) with CRP results of ≤2.5 mg/L were erroneously reported as exactly 2.5 mg/L. We performed sensitivity analyses (1) excluding these samples and (2) assigning midpoint values (between 0 and 2.5 mg/L) of 1.25 mg/L. “Near treatment end” was defined as completing ≥4 months of TB treatment.

The performance of CRP for TB diagnosis was evaluated using 5 and 10 mg/L cutoffs [8]. Correlates of CRP positivity (≥5 mg/L) were assessed using generalized linear models with log link and Poisson family. Statistical significance was assessed using χ^2^ tests for categorical variables and Wilcoxon rank sum tests for continuous variables. Serial CRP was compared between baseline and near treatment end using McNemar χ^2^ and signed rank tests. Stratified analysis examined CRP treatment response by baseline CRP level (<5 vs ≥5 mg/L).

We estimated the accuracy of CRP for TB diagnosis by calculating the area under the curve (AUC) for receiver operating characteristic (ROC) analysis, comparing (1) children with confirmed TB and those with unlikely TB; (2) children with confirmed TB and those with unconfirmed or unlikely TB; and (3) children with confirmed or unconfirmed TB and those with unlikely TB. Cutoffs were determined using the Youden index for optimal sensitivity-specificity balance, with additional analysis targeting 90% sensitivity.

### Ethical Considerations

This study was approved by the Kenya Medical Research Institute Scientific Ethics Research Unit, the University of Washington Institutional Review Board, and the University of Nairobi/Kenyatta National Hospital Ethics and Research Committee. We obtained parental consent for all enrolled children and assent from those aged ≥13 years.

## RESULTS

Between May 2022 and July 2023, a total of 467 children were screened for eligibility, 330 were enrolled, and 292 (88.5%) with CRP results were included in the analysis; 38 lacked CRP results due to insufficient blood volume and were excluded from this analysis (Supplementary Figure 1). Their median age was 3.0 years (interquartile range, 1.0–5.0 years), 46.2% were male (n = 135), and 3.1% (n = 9) were living with HIV (Supplementary Table 1). Eighteen children (6.2%) had confirmed, 183 (62.7%) had unconfirmed, and 87 (29.8%) had unlikely TB, and 4 (1.4%) were unclassifiable. A detailed description of clinical presentation and comprehensive diagnostic workup completion is provided in Supplementary Tables 2 and 3. Baseline CRP levels did not differ significantly in children with confirmed (median, 3.8 mg/L; *P* = .11), unconfirmed (1.8 mg/L; *P* = .25), or unclassifiable (4.4 mg/L; *P* = .20) TB compared with those with unlikely TB (median, 1.6 mg/L) (Table 1).

**Table 1.** Screening Performance of C-Reactive Protein in Pulmonary Tuberculosis (TB) and Treatment Response Monitoring^a^

				
CRP Level	Confirmed TB^b^	Unconfirmed TB^b^	Unlikely TB^b^	Unclassifiable
Diagnostic screening (N = 292)	(n = 18)	(n = 183)	(n = 87)	(n = 4)
CRP ≥5 mg/L, no. (%)	9 (50.0)	65 (35.5)	25 (28.7)	2 (50)
* P* value^c^	.08	.27	Reference	.36
CRP ≥10 mg/L, no. (%)	8 (44.4)	52 (28.4)	21 (24.1)	1 (25)
* P* value^c^	.08	.46	Reference	.97
CRP, median (IQR), mg/L	3.8 (0.5–47.8)	1.8 (0.5–13.4)	1.6 (0.3–8.4)	4.4 (2.1–18.9)
* P* value^c^	.11	.25	Reference	.20
Treatment response^d^ (n = 97)	n = 11	n = 86	…	…
CRP before TB treatment, median (IQR), mg/L	8.1 (0.4–54.0)	2.4 (0.7–12.8)	…	…
CRP near treatment end, median (IQR), mg/L)	0.5 (0.2–1.2)	0.8 (0.3–2.5)	…	…
* P* value^e^	.02	<.001	…	…

Abbreviations: CRP, C-reactive protein; IQR, interquartile range; TB, tuberculosis.

^a^Median values were compared using Wilcoxon signed rank sum tests, and sensitivity/specificity values using McNemar χ^2^ tests.

^b^Based on international consensus clinical case definitions for pediatric TB with post hoc classification using Graham's criteria [7].

^c^Confirmed, unconfirmed, or unclassifiable TB compared with unlikely TB.

^d^Among participants who completed ≥4 months of TB treatment. (No participants in the unlikely TB group received TB treatment.)

^e^Before TB treatment compared with near treatment end.

At a 5 mg/L threshold, the sensitivity of CRP was 50.0% (95% confidence interval [CI], 26.0%–74.0%) for confirmed and 35.5% (29.0%–42.4%) for unconfirmed TB, with 71.3% (60.4%–80.6%) specificity for unlikely TB (Table 1). At the 10 mg/L threshold, the sensitivity decreased to 44.4% (95% CI, 21.5%–69.2%) for confirmed and 28.4% (22.2%–35.4%) for unconfirmed TB, with 75.9% (65.5%–84.4%) specificity among children with unlikely TB.

**Figure 1.**
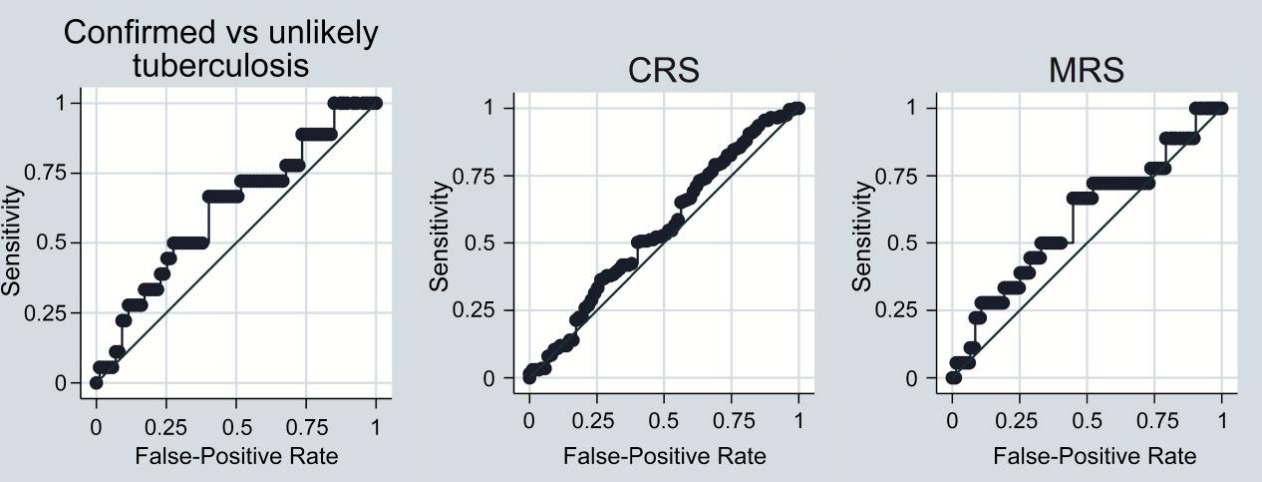
Receiver operating characteristic (ROC) curves for C-reactive (CRP) protein in tuberculosis (TB) diagnosis. Three ROC curves compare CRP diagnostic performance using different reference standards: confirmed versus unlikely TB (area under the curve [AUC], 0.62), composite reference standard (CRS) with confirmed plus unconfirmed versus unlikely TB (AUC, 0.55), and microbiological reference standard (MRS) with confirmed versus unconfirmed plus unlikely TB (AUC, 0.59).

ROC analysis showed that the AUC was 0.62 (95% CI, .48–.76) for confirmed TB versus unlikely TB, with a cutoff value of 2.33 mg/L. For the microbiologic reference standard (confirmed vs unconfirmed + unlikely TB), the AUC was 0.59 (95% CI, .45–.74), with a cutoff of 2.44 mg/L. For the composite reference standard (confirmed + unconfirmed vs unlikely TB), the AUC was 0.55 (95% CI, .48–.63) with a cutoff of 0.44 mg/L (Figure 1 and Supplementary Table 4). Analysis prioritizing 90% sensitivity yielded impractical cutoffs (≤0.3 mg/L).

Among 97 children who completed ≥4 months of TB treatment, the median CRP level was higher before treatment than to near the end of treatment among children with confirmed (8.1 vs 0.5 mg/L; *P* = .02) or unconfirmed (2.4 vs 0.8 mg/L; *P* < .001) TB (Table 1). CRP levels declined progressively during treatment (Supplementary Table 5). Sensitivity analysis (1) excluding 57 participants with erroneously reported CRP values of 2.5 mg/L for values of 0–2.5 mg/L and (2) setting them at 1.25 mg/L yielded similar results, with no impact on study findings (Supplementary Table 6).

Stratified analysis revealed that CRP decline during treatment occurred primarily in children with baseline elevation ≥5 mg/L (40% of children), while those with baseline CRP <5 mg/L showed minimal change (Supplementary Table 7). CRP decline showed weak correlations with body mass index improvement and symptom resolution (Supplementary Table 8). No unlikely TB participants received antituberculosis treatment, and alternative diagnoses were identified through routine clinical care.

Baseline positive QuantiFERON Gold test (QFT) was associated with 1.5 times higher risk of baseline CRP positivity. Children living with HIV had double the risk of CRP positivity. Chest radiographs suggestive of TB, confirmed TB, and TB treatment initiation were associated with increased CRP positivity risk in univariate analysis, but these associations were not statistically significant after adjustment (Supplementary Table 9). Among children with unconfirmed TB, CRP elevation did not differ significantly between children in higher-probability and lower-probability TB groups (Supplementary Table 10).

## DISCUSSION

While screening diagnostic performance of CRP was suboptimal in children, CRP levels significantly decreased during TB treatment in those with confirmed or unconfirmed TB, suggesting potential targeted utility for treatment response monitoring. Among adults with TB, results have generally met the WHO criteria for a screening test with ≥90% sensitivity and ≥70% specificity, particularly in people living with HIV where WHO endorsement exists [4, 5]. However, our study's sensitivity did not meet WHO criteria at the 5 or 10 mg/L thresholds, consistent with prior pediatric studies [4, 5], highlighting a performance divergence for TB diagnosis between children and adults.

Stratified analysis demonstrated that CRP decline during TB treatment occurred primarily in the subset of children with baseline elevation ≥5 mg/L (40%). This pattern mirrors findings from adult cohorts showing that CRP decreases with effective treatment [9, 10], suggesting targeted use in pediatric TB treatment monitoring. Consistent with a Ugandan study that enrolled symptomatic children [5], ROC analysis revealed moderate diagnostic performance (AUC, 0.62 for confirmed vs unlikely TB and 0.59 for microbiologic reference standard), although the composite reference standard showed near-random performance (AUC, 0.55).

Strengths of the current study include comprehensive investigations using consensus TB classification guidelines, standardized follow-up, and stratified analysis providing novel guidance for targeted biomarker use in real-world pediatric TB scenarios. Study limitations include potential referral bias due to a high proportion of unconfirmed TB (62.7%), which may limit generalizability for community screening but reflects common pediatric clinical presentations. The small number of children with HIV (n = 9 [3.1%]) limits analysis given the established utility of CRP in HIV-positive adults. Treatment monitoring findings were limited to participants who initiated therapy, precluding comparison with untreated cases.

In conclusion, CRP did not meet WHO triage targets for pediatric TB diagnosis, confirming its limited utility as a screening tool. However, CRP levels declined during treatment among children with elevated baseline levels, supporting targeted use for treatment monitoring. Future work should evaluate CRP as part of multimodal treatment monitoring strategies rather than as a diagnostic tool.

## Supplementary Material

ofaf816_Supplementary_Data

## Supplementary Data

Supplementary materials are available at *Open Forum Infectious Diseases* online. Consisting of data provided by the authors to benefit the reader, the posted materials are not copyedited and are the sole responsibility of the authors, so questions or comments should be addressed to the corresponding author.
