# Supplementary material for: C-Reactive Protein for Pulmonary Tuberculosis Screening and Treatment Response Monitoring in Children
Source: Open Forum Infect Dis. 2026 Jan 7;13(2):ofaf816. doi: 10.1093/ofid/ofaf816 (PMC12872367; doi:10.1093/ofid/ofaf816)
Supplement: ofaf816_Supplementary_Data [file ofaf816_supplementary_data.docx]

**Supplemental Figure 1: Participant Flowchart**

**
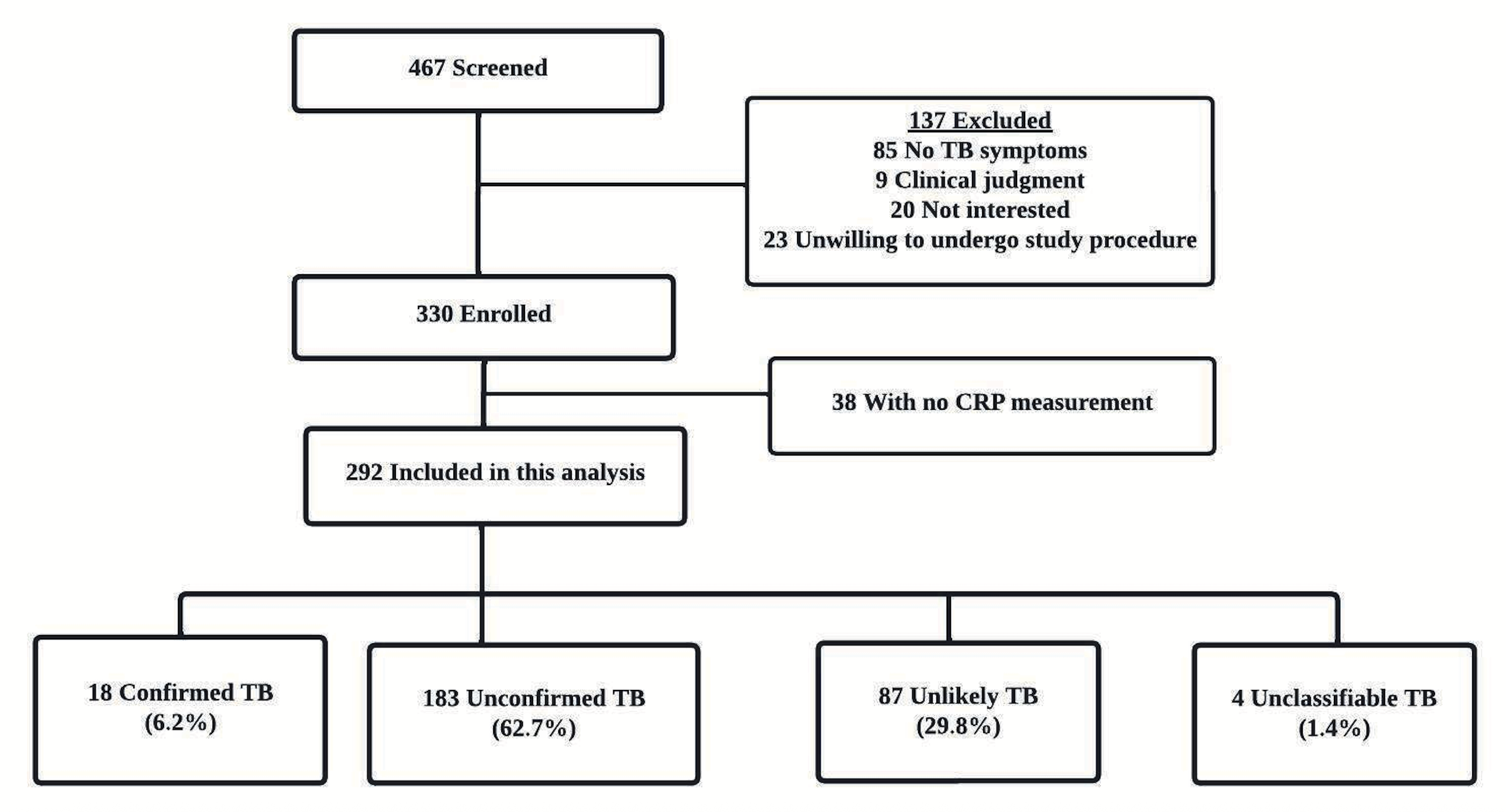
**

**Supplemental Figure 1:** Study participant flowchart depicting the selection and classification process. Beginning with 467 children screened for tuberculosis symptoms, 137 were excluded for the following reasons: 85 had no TB symptoms, 9 were excluded based on clinical judgment, 20 were not interested in participating, and 23 were unwilling to undergo study procedures. This left 330 children enrolled in the study. Of these, 38 were excluded due to missing CRP measurements due to insufficient blood volume, resulting in 292 children included in the final analysis. The 292 participants were classified into four diagnostic categories: 18 children with confirmed TB representing 6.2% of the cohort, 183 children with unconfirmed TB representing 62.7%, 87 children with unlikely TB representing 29.8%, and 4 children who were unclassifiable representing 1.4% of the study population.

**Supplemental Table 1: Baseline Characteristics**

|  | **Overall** | **Confirmed TB ^a^** | **Unconfirmed TB^a^** | **Unlikely TB^a^** | **Unclassifiable^a^** | |
| --- | --- | --- | --- | --- | --- | --- |
|  | **N=292** | **N=18** | **N=183** | **N=87** | **N=4** | |
|  | **Median (IQR) or n (%)** | **Median (IQR) or n (%)** | **Median (IQR) or n (%)** | **Median (IQR) or n (%)** | **Median (IQR) or n (%)** | |
| **Demographics** | | | | | |  |
| Age (years) | 3.0 (1.0, 5.0) | 1.5 (1.0, 8.0) | 2.0 (1.0, 5.0) | 3.0 (1.0, 8.0) | 1.5 (0.5, 3.0) | |
| Female sex | 157 (53.8) | 11 (61.1) | 96 (52.5) | 47 (54.0) | 3 (75.0) | |
| **Clinical Presentation** | | | | | |  |
| BMIz (N=289) | -0.4 (-1.8, 0.5) | -0.7 (-1.2, -0.0) | -0.5 (-2.0, 0.6) | -0.3 (-1.6, 0.5) | -0.2 (-1.9, 0.5) | |
| Underweight^b^  (BMIz<-2) (N=289) | 64 (22.1) | 2 (11.1) | 44 (24.2) | 17 (20.0) | 1 (25.0) | |
| WHZ (N=203)**^c^** | -0.7 (-2.1, 0.3) | -0.8 (-1.4, -0.4) | -0.5 (-2.3, 0.5) | -0.8 (-2.1, 0.3) | -0.4 ( -2.1,0.3) | |
| Wasted (WHZ<-2) (N=203)**^d^** | 58 (28.6) | 2 (16.7) | 40 (30.1) | 15 (27.8) | 1 (25.0) | |
| WAZ (N=204)^c^ | -1.4 (-2.4, -0.4) | -1.9 (-2.6, -0.6) | -1.3 (-2.5, -0.3) | -1.3 (-2.3, -0.4) | -1.6 (-2.5, -1.0) | |
| Underweight  (WAZ<-2) (N=204)**^d^** | 73 (35.8) | 5 (41.7) | 49 (36.6) | 18 (33.3) | 1 (25.0) | |
| CLHIV | 9 (3.1) | 0 (0.0) | 9 (4.9) | 0 (0.0) | 0 (0.0) | |
| **TB Features** | | | | | |  |
| NIH criteria signs/symptoms of TB **^e^** | 238 (81.5) | 16 (88.9) | 160 (87.4) | 60 (69.0) | 2 (50.0) | |
| TST positive (N=288) | 104 (36.1) | 10 (55.6) | 71 (39.7) | 21 (24.1) | 2 (50.0) | |
| QFT Positive (N=222) | 36 (12.3) | 5 (27.8) | 22 (12.0) | 9 (10.3) | 0 (0.0) | |
| TB contact | 117 (40.1) | 10 (55.6) | 76 (41.5) | 31 (35.6) | 0 (0.0) | |
| CXR suggestive of TB | 185 (63.4) | 16 (88.9) | 137 (74.9) | 32 (36.8) | 0 (0.0) | |
| Culture positive(N=261) **^f^** | 9 (3.4) | 9 (52.9) | -- | -- | -- | |
| Xpert positive(N=276) **^f^** | 15 (5.4) | 15 (83.3) | -- | -- | -- | |
| Urine LAM positive (N=166) | 17 (10.2) | 1 (10.0) | 9 (8.9) | 7 (13.5) | 0 (0.0) | |
| TBtx-initiated **^g^** | 127 (43.5) | 15 (83.3) | 112 (61.2) | 0 (0.0) | 0 (0.0) | |
| Positive response TBtx (N=127) **^h^** | 116 (91.3) | 13 (86.7) | 103 (92.0) | 0 (0.0) | 0 (0.0) | |
| **Abbreviations**: IQR: Interquartile range; WAZ ,weight for-age z score; WHZ, weight for-height z score; BMI, Body Mass Index (BMI) –for-age z score ; HUU, HIV unexposed uninfected; HEU, HIV exposed uninfected; CLHIV, children living with HIV; NIH, National Institutes of Health; TST, tuberculin skin test; QFT, QuantiFERON test; CXR, chest radiograph; Mtb, mycobacterium tuberculosis; Xpert, Xpert MTB/ULTRA; LAM, lipoarabinomannan; TBtx,TB treatment.  **N:** Number of participants with results; **n:** number of participants with positive results  ^a^ Based on international consensus clinical case definitions for pediatric TB via post-hoc classification.  ^b^  BMI z<-2 For the entire population  ^c^ Among children 5 years and under  ^d^ Among children 5 years and under: WHZ <-2 or MUAC <12·5 cm or WAZ<-2 r  ^e^ Persistent cough (>14 days), fever (>7 days), failure to thrive, or lethargy (>7 days)· Failure to thrive=wasted (WHZ<-2 or MUAC<12·5) or underweight (WHZ<-2) or Thinness (BMI <-2) at enrollment (growth trajectories unavailable before enrollment). ^f^ Sputum or gastric aspirate.  ^g^ Received TB treatment at enrollment or 2-weeks after enrollment  ^h^ Positive response to TB treatment after 2 weeks of enrollment | | | | | | |

**Supplemental Table 2: Detailed Clinical Characteristics by TB Classification**

| **Characteristic** | **Confirmed TB^a^**  **N=18**  **n (%) or median (IQR)** | **Unconfirmed TB^a^**  **N=183**  **n (%) or median (IQR)** | **Unlikely TB^a^**  **N=87**  **n (%) or median (IQR)** | **Unclassifiable^a^**  **N=4**  **n (%) or median (IQR)** | **P-value^b^** |  |
| --- | --- | --- | --- | --- | --- | --- |
| **Respiratory Symptoms** |  |  |  |  |  |  |
| Cough present | 18 (100) | 175 (95.6) | 80 (92.0) | 4 (100) | 0.40 |  |
| Persistent cough >14 days | 15 (83.3) | 143 (78.1) | 54 (62.1) | 2 (50) | <0.020 |  |
| Cough duration, median days (IQR)^d^ | 17.5 (14.0, 30.0) | 21.0 (14.0, 30.0) | 16.5 (7.0, 30.0) | 10.5 (6.5, 37.0) | 0.32 |  |
| **Constitutional Symptoms** |  |  |  |  |  |  |
| Fever present | 9 (50.0) | 102 (55.7) | 43 (49.4) | 3 (75.0) | 0.87 |  |
| Fever >7 days | 8 (44.4) | 71 (38.8) | 21 (24.1%) | 0 (0) | 0.036 |  |
| Fever duration, median days (IQR)^e^ | 14.0 (7.0,21.0) | 7.0 (4.0,14.0) | 5.0 (3.0,14.0) | 2.0 (1.0,3.0) | 0.004 |  |
| Night sweats (drenching) | 8 (44.4) | 105 (57.4) | 41 (47.1) | 0 (0) | 0.13 |  |
| Weight loss/poor weight gain | 9 (50.0) | 106 (57.9) | 51 (58.6) | 2 (50.0) | 0.96 |  |
| Lethargy/fatigue | 3 (16.7) | 29 (15.8) | 12 (13.8) | 0 (0) | 0.82 |  |
| Decreased appetite | 10 (55.6) | 78 (42.6) | 38 (43.7) | 2 (50) | 0.76 |  |
| **Physical Examination** |  |  |  |  |  |  |
| Cervical lymphadenopathy | 1 (5.6) | 17 (19.3) | 11 (12.6) | 1 (25.0) | 0.55 |  |
| **Laboratory parameters** |  |  |  |  |  |  |
| **Hemoglobin status** |  |  |  |  |  |  |
| Anemia | 5 (27.8) | 59 (33.7) | 17 (20.2) | 1 (25) | 0.17 |  |
| QFT positive | 5 (27.8) | 22 (12.0) | 9 (10.3%) | 0 (0) | 0.42 |  |
| **Supporting Evidence** |  |  |  |  |  |  |
| Past TB exposure | 10 (55.6) | 76 (41.5) | 31 (35.6) | 0 (0) | 0.15 |  |
| CXR suggestive of TB | 16 (88.9) | 137 (74.9) | 32 (36.8) | 0 (0) | <0.001 |  |
| **Abbreviations:** CRP, C-reactive protein; CXR, chest X-ray; IQR, interquartile range; QFT, QuantiFERON Gold test; TB, tuberculosis.  ^a^ Based on international consensus clinical case definitions for pediatric TB via post-hoc classification using Graham's criteria.  ^b^ P-values from chi-square test for categorical variables and Wilcoxon rank-sum test for continuous variables.  ^c^ Anemia defined as hemoglobin levels <11.1 g/dL for all children  ^d^ Among participants with a cough present.  ^e^ Among participants with a fever present.  Values presented as n (%) for categorical variables and median (IQR) for continuous variables. | | | | | | |

**Supplemental Table 3: Diagnostic Workup Completion by TB Classification**

| **Test** | **Confirmed TB^a^**  **N=18 (%)** | **Unconfirmed TB^a^**  **N=183 (%)** | **Unlikely TB^a^**  **N=87 (%)** | **Unclassifiable^a^**  **N=4 (%)** |
| --- | --- | --- | --- | --- |
| Gene Xpert | 18 (100) | 173 (94.5) | 81 (93.1) | 4 (100) |
| Smear microscopy | 18 (100) | 172 (94.0) | 82 (94.3) | 3 (75.0) |
| Culture | 18 (100) | 168 (91.8) | 82 (94.3) | 3 (75.0) |
| Chest X-ray | 18 (100) | 183 (100) | 87 (100) | 4 (100) |
| TST (Tuberculin Skin Test) | 18 (100) | 183 (100) | 87 (100) | 4 (100) |
| QFT (QuantiFERON Gold) Plus | 17 (94.4) | 153 (83.6) | 76 (87.4) | 3 (75.0) |
| Urine LAM test | 10 (55.6) | 102 (55.7) | 52 (59.8) | 3 (75.0) |
| Complete Blood Count | 18 (100) | 177 (96.0) | 84 (96.6) | 4 (100) |
| **Abbreviations:** LAM, lipoarabinomannan; QFT, QuantiFERON Gold test; TB, tuberculosis; TST, tuberculin skin test.  ^a^ Based on international consensus clinical case definitions for pediatric TB via post-hoc classification using Graham's criteria. Values presented as n (%) of participants in each TB classification group who received the specified diagnostic test. Percentages calculated based on total participants per group. | | | | |

**Supplemental Table 4: Area Under the ROC Curve for C-Reactive Protein Levels to Detect Childhood TB**

| **Reference Standard** | **AUC (95% CI)** | **Cut-off mg/L^a^** | **Sensitivity % (95%CI)** | **Specificity % (95%CI)** | **PPV** | **NPV** |
| --- | --- | --- | --- | --- | --- | --- |
| **Confirmed vs. Unlikely** | 0.62  (0.48, 0.76) | 2.33 | 66.7  (41.0, 86.7) | 59.8  (48.7, 70.1) | 25.5  (13.9, 40.3) | 89.7  (78.8, 96.1) |
| **MRS** | 0.59  (0.45, 0.74) | 2.44 | 66.7  (41.0, 86.7) | 55.2  (49.0, 61.2) | 9.0  (4.7, 15.2) | 96.1  (91.8, 98.6) |
| **CRS** | 0.55  (0.48, 0.63) | 0.44 | 79.1  (72.8, 84.5) | 31.0  (21.5, 41.9) | 72.6  (66.2, 78.4) | 39.1  (27.6, 51.6) |
| **Abbreviations :** Receiver operating characteristic **(**ROC); Area under the ROC (AUC) ; Positive predictive value (PPV)  Negative predictive value (NPV) ; Composite reference standard (CRS)= Confirmed + Unconfirmed vs. Unlikely TB,  Microbiological reference standard (MRS)= Confirmed vs. Unconfirmed + Unlikely  ^a^ Optimal cut-off for each of the reference standards | | | | | | |

**Supplemental Table 5: Participant CRP Values Through Follow-up**

| **Timepoint** | **Confirmed TB (N), median (IQR)** | **Unconfirmed TB (N), median (IQR)** | **Unlikely TB (N), median (IQR)** | **Unclassifiable (N), median (IQR)** |
| --- | --- | --- | --- | --- |
| Enrollment (N=292) | 18, 3.8 (0.5,47.8) | 183, 1.8 (0.5,13.4) | 87, 1.6 (0.3,8.4) | 4, 4.4 (2.1, 18.9) |
| 1-month follow-up (N=100) | 7, 1.9 (0.4,8.1) | 82, 0.5 (0.2,2.5) | 11, 0.4 (0.2,2.5) | - |
| 4-month follow-up (N=120) | 10, 0.3 (0.2,0.6) | 102, 0.5 (0.2,1.6) | 8, 0.4 (0.2,0.8) | - |
| 6-month follow-up (N=99) | 8, 0.9 (0.5,1.2) | 89, 1.2 (0.4,2.5) | 2, 2.7 (2.5,2.9) | - |
| **Abbreviations:** CRP, C-reactive protein; IQR, interquartile range; TB, tuberculosis.  Values presented as median (IQR) mg/L. Sample sizes (N) indicate number of participants with available CRP measurements at each timepoint. Follow-up timepoints: 1-month (±2 weeks), 4-month (±4 weeks), 6-month (±4 weeks). | | | | |

**Supplemental Table 6: Sensitivity Analysis of Screening Performance of CRP in Pulmonary TB and Treatment Response Monitoring for samples (n=57) from 54 participants) erroneously reported as 2.5 mg/L for values of 0-2.5 mg/L**

| **Diagnostic screening: Setting to 1.25mg/L midpoint^¥^** | | | | |
| --- | --- | --- | --- | --- |
| **N=292** | **Confirmed TB^a^**  **n=18** | **Unconfirmed TB^a^ n=183** | **Unlikely TB^a^**  **n=87** | **Unclassifiable^a^**  **n=4** |
| CRP (Median, IQR) | 3.8 (0.5, 47.8) | 1.3 (0.5, 13.4) | 1.3 (0.3, 8.4) | 4.4 (2.1,18.9) |
| P-Value* | 0.13 | 0.31 | Reference | 0.17 |
| **Diagnostic screening: Exclusion of 2.5mg/L^#^** | | | |  |
| **N=280** | **Confirmed TB^a^**  **n=17** | **Unconfirmed TB^a^ n=174** | **Unlikely TB^a^**  **n=85** | **Unclassifiable TB^a^**  **n=4** |
| CRP ≥5 mg/L | 9 (52.9 %) | 65 (37.4 %) | 25 (29.4 %) | 2 (50%) |
| P-Value* | 0.06 | 0.21 | Reference | 0.38 |
| CRP ≥10 mg/L | 8 (47.1 %) | 52 (29.9%) | 21 (24.7 %) | 1 (25%) |
| P-Value* | 0.06 | 0.38 | Reference | 0.99 |
| CRP (Median, IQR) | 5.1 (0.5, 47.8) | 1.4 (0.5, 13.8) | 1.5 (0.3, 8.4) | 4.4 (2.1,18.9) |
| P-Value* | 0.12 | 0.27 | Reference | 0.19 |
| **Treatment Response**: Setting to 1.25mg/L^¥^** | | | | |
| **N=97** | **Confirmed TB^a^**  **n=11** | **Unconfirmed TB^a^ n=86** | **Unlikely TB^a^** | **Unclassifiable** |
| Pre TBtx-CRP mg/L (Median, IQR) | 8.1 (0.4, 54.0) | 1.5 (0.7, 12.8) | -- | -- |
| Near TBtx end CRP mg/L (Median, IQR) | 0.5 (0.2, 1.2) | 0.8 (0.3, 1.2) | -- | -- |
| P-Value*** | **0.02** | **<0.001** | -- | -- |
| **Treatment Response**: Diagnostic screening: Exclusion of 2.5mg/L^#^** | | | | |
| **N=77** | **Confirmed TB^a^**  **n=10** | **Unconfirmed TB^a^ n=67** | **Unlikely TB^a^** | **Unclassifiable^a^** |
| Pre TBtx-CRP mg/L (Median, IQR) | 10.0 (0.4, 54.0) | 1.8 (0.7, 17.3) | -- | -- |
| Near TBtx end CRP mg/L (Median, IQR) | 0.6 (0.2, 1.2) | 0.7 (0.3, 1.6) | -- | -- |
| P-Value*** | **0.04** | **0.0001** | -- | -- |
| **Abbreviations**: IQR, Interquartile range; TB, tuberculosis; TBtx, TB treatment  ^a^ Based on international consensus clinical case definitions for pediatric TB via post-hoc classification.  * Confirmed or Unconfirmed TB compared to Unlikely TB  ** Among participants who completed at least 4 months of TB treatment. No children with Unlikely or Unclassifiable TB met inclusion criteria for this analysis.  *** Pre TB treatment compared to near treatment end  ¥- Setting CRP levels inadvertently reported as 2.5mg/L to 1.25mg/L  # Excluding participants whose result was inadvertently reported as 2.5mg/L for levels of 2.5 mg/L  Median values were compared using Wilcoxon signed rank-sum test  Sensitivity/specificity comparisons were done using Mc-Nemar’s chi square test | | | | |

**Supplemental Table 7:** **CRP Changes at Follow-up by Baseline CRP Level**

| **TB Classification** | **Baseline CRP** | **Follow-up <5 mg/L** | **Follow-up ≥5 mg/L** | **Total** |
| --- | --- | --- | --- | --- |
| **Unconfirmed TB** | <5 mg/L | 47 | 7 | 54 |
|  | ≥5 mg/L | 31 | 1 | 32 |
|  | **Total** | **78** | **8** | **86** |
| **Confirmed TB** | <5 mg/L | 4 | 0 | 4 |
|  | ≥5 mg/L | 5 | 2 | 7 |
|  | **Total** | **9** | **2** | **11** |
| Values represent number of participants with paired baseline and follow-up CRP measurements (n=97). Follow-up measurements taken at ≥4 months of TB treatment (near treatment end). | | | | |

**Supplemental Table 8: Clinical Severity and Recovery Measures**

| **Parameter** | **Confirmed TB^a^**  **n=18** | **Unconfirmed TB^a^**  **n=183** | **Unlikely TB^a^**  **n=87** | **Unclassifiable^a^**  **n=4** | **P-value** |  |
| --- | --- | --- | --- | --- | --- | --- |
| **Growth Parameters** |  |  |  |  |  |  |
| BMI-for-age Z-score |  |  |  |  |  |  |
| Baseline (N=289) | (N=18) -0.7 (-1.2,-0.0) | (N=182) -0.5 (-2.0,0.6) | (N=85) -0.3 (-1.6,0.5) | (N=4) -0.2 (-1.9,0.5) | 0.77 |  |
| 6 months (N=99) | (N=8) 0.3 (-0.0,0.4) | (N=89) 0.3 (-0.6,0.9) | (N=2) -2.0 (-3.5,-0.5) |  | 0.19 |  |
| P-value (baseline to 6 months) | 0.02 (n=7) | <0.001 (n=84) | 1.00 (n=1) |  |  |  |
| **Presence of TB Symptoms** |  |  |  |  |  |  |
| Baseline (292) | 16 (88.9) | 160 (87.4) | 62 (71.3) | 2 (50.0) | 0.003 |  |
| Month 4 (N=120) | 0 (0) | 17 (16.7) | 2 (25.0) |  | 0.30 |  |
| Month 6 (N=99) | 0 (0) | 17 (19.1) | 1 (50.0) |  | 0.20 |  |
| **Laboratory Parameters** |  |  |  |  |  |  |
| Hemoglobin (g/dL) baseline (N=281) | (N=18) 12.0 (10.8,12.9) | (N=175) 11.8 (10.8,12.9) | (N=84) 12.7 (11.4,13.5) | (N=4) 12.0 (10.3,12.4) | - |  |
| **Correlation coefficient (r) HB with CRP at baseline** | 0.048 | -0.213 | -0.119 | -0.941 |  |  |
| **Correlation coefficient (r) CRP percent decline vs. BMI improvement** | -0.0580 | 0.0005 | - |  |  |  |
| **Abbreviations:** BMI, body mass index; TB, tuberculosis.  ^a^ Based on international consensus clinical case definitions for pediatric TB via post-hoc classification  Values presented as median (IQR) for continuous variables and n (%) for categorical variables.  Correlation coefficients calculated within each TB classification group using the n shown in column headers.  P-values calculated using paired t-test for within-group changes and ANOVA for between-group comparisons. | | | | | | |

| **Supplemental Table 9: Correlates of CRP positivity at baseline (CRP ≥5 mg/L)** | | | | | | | | |
| --- | --- | --- | --- | --- | --- | --- | --- | --- |
| **Baseline characteristics** | **Overall** | **CRP positive**  **N=101**  **n (%) or median (IQR)** | **CRP negative**  **N=191**  **n (%) or median (IQR)** | **RR (95% CI)^*^** | **p** | **aRR (95% CI)^¥^** | **p** |  |
|  | **N=292** |  |  |  |  |  |  |  |
|  | **n (%) or median (IQR)** |  |  |  |  |  |  |  |
| **Demographics** | | | | | | | | |
| Age (years) | 3.0 (1.0, 5.0) | 3.0 (1.0, 5.0) | 2.0 (1.0, 5.0) | 1.01 (0.97-1.05) | 0.636 | - | - |  |
| Female sex | 157 (53.8) | 59 (58.4) | 98 (51.3) | 1.21 (0.87-1.67) | 0.251 | - | - |  |
| **Clinical Presentation** | | | | | | | | |
| BMIz (N=289) | -0.4 (-1.8, 0.5) | -0.7 (-2.0, 0.3) | -0.3 (-1.7, 0.5) | 0.94 (0.87-1.02) | 0.114 | - | - |  |
| Underweight^a^  (BMIz<-2) (N=289) | 64 (22.1) | 26 (25.7) | 38 (20.2) | 1.22 (0.86-1.73) | 0.268 | - | - |  |
| WHZ (N=204)^b^ | -0.7 (-2.1, 0.3) | -0.8 (-2.3, 0.3) | -0.5 (-2.1, 0.4) | 0.97 (0.88-1.08) | 0.560 | - | - |  |
| Wasted (WHZ<-2) (N=203)^c^ | 58 (28.6) | 21 (30.4) | 37 (27.6) | 1.09 (0.72-1.65) | 0.671 | - | - |  |
| WAZ (N=204) ^b^ | -1.4 (-2.4, -0.4) | -1.7 (-2.6, -0.4) | -1.2 (-2.3, -0.4) | 0.94 (0.83-1.07) | 0.382 | - | - |  |
| Underweight  (WAZ<-2)(N=204)^c^ | 73 (35.8) | 29 (42.0) | 44 (32.6) | 1.30 (0.89-1.91) | 0.179 | - | - |  |
| HIV status |  |  |  |  |  |  |  |  |
| HUU | 275 (94.2) | 92 (91.1) | 183 (95.8) | Reference |  | Reference |  |  |
| HEU | 8 (2.7) | 3 (3.0) | 5 (2.6) | 1.12 (0.45-2.79) | 0.806 | 1.24 (0.56-2.76) | 0.600 |  |
| CLHIV | 9 (3.1) | 6 (5.9) | 3 (1.6) | 1.99 (1.22-3.26) | **0.006** | 1.97 (1.18-3.27) | **0.009** |  |
| **TB Features** | | | | | | | | |
| NIH criteria signs/symptoms of TB^d^ | 238 (81.5) | 80 (79.2) | 158 (82.7) | 0.86 (0.59-1.26) | 0.452 |  |  |  |
| TB Classification |  |  |  |  |  |  |  |  |
| Confirmed TB | 18 (6.2) | 9 (8.9) | 9 (4.7) | 1.74 (0.98-3.07) | **0.057** | 1.13 (0.58-2.20) | 0.715 |  |
| Unconfirmed TB | 183 (62.7) | 65 (64.4) | 118 (61.8) | 1.24 (0.84-1.82) | 0.280 | 0.89 (0.53-1.48) | 0.642 |  |
| Unclassifiable | 4 (1.4) | 2 (2.0) | 2 (1.0) | 1.74 (0.62-4.90) | 0.295 | 2.06 (0.75-5.69) | 0.162 |  |
| Unlikely TB | 87 (29.8) | 25 (24.8) | 62 (32.5) | Reference |  | Reference |  |  |
| TST positive (N=288) | 104 (36.1) | 31 (31.3) | 73 (38.6) | 0.81 (0.57-1.15) | 0.230 |  |  |  |
| QFT Positive (N=222) | 36 (12.3) | 17 (16.8) | 19 (9.9) | 1.51 (1.01-2.27) | **0.046** | 1.55 (1.02-2.35) | **0.040** |  |
| TB contact | 117 (40.1) | 38 (37.6) | 79 (41.4) | 0.90 (0.65-1.25) | 0.539 |  |  |  |
| CXR suggestive of TB | 185 (63.4) | 71 (70.3) | 114 (59.7) | 1.37 (0.96-1.95) | **0.083** | 1.33 (0.89-1.99) | 0.171 |  |
| Mtb culture/ Xpert positive(N=276)^e^ | 18 (6.5) | 9 (9.5) | 9 (5.0) | 1.50 (0.92-2.46) | 0.108 |  |  |  |
| Urine LAM positive(N=166) | 17 (10.2) | 3 (5.7) | 14 (12.4) | 0.53 (0.18-1.51) | 0.232 |  |  |  |
| TBTx initiated^f^ | 127 (43.5) | 53 (52.5) | 74 (38.7) | 1.43 (1.05-1.97) | **0.025** | 1.30 (0.85 -1.99) | 0.224 |  |
| Positive response TBTx (N=127^g^ | 116 (91.3) | 51 (96.0) | 65 (88.0) | 2.42 (0.68-8.66) | 0.175 |  |  |  |
| **Abbreviations:** IQR: Interquartile range; WAZ ,weight for-age z score; WHZ, weight for-height z score; BMI, Body Mass Index (BMI) –for-age z score ; HUU, HIV unexposed uninfected; HEU, HIV exposed uninfected; CLHIV, children living with HIV; NIH, National Institutes of Health; TST, tuberculin skin test; QFT, QuantiFERON test; CXR, chest radiograph; Mtb, mycobacterium tuberculosis; Xpert, Xpert MTB/ULTRA; LAM, lipoarabinomannan; TBTx, TB treatment.  N: Number of participants with results; n: number of participants with positive results  ^a^ BMIz<-2 For the entire population  ^b^ Among children 5 years and under  ^c^ Among children 5 years and under: WHZ <-2 or MUAC <12·5 cm or WAZ<-2  ^d^ Persistent cough (>14 days), fever (>7 days), failure to thrive, or lethargy (>7 days)· Failure to thrive=wasted (WHZ<-2 or MUAC<12·5) or underweight (WHZ<-2) or Underweight (BMI <-2) at enrollment (growth trajectories unavailable before enrollment). ^e^ Sputum or gastric aspirate.  ^f^ Received TB treatment at enrollment or 2-weeks after enrollment  ^g^ Positive response to TB treatment after 2 weeks of enrollment  *Relative risk (RR) estimated using a generalized linear model (GLM) with log link and Poisson family  **^¥^** Adjusted for CXR in multivariate regression, including variables with p<0.1 from univariate analysis | | | | | | | | |

**Supplemental Table 10: Unconfirmed TB Stratified by Clinical Probability Score**

| **Characteristic** | **Higher Probability^a^**  **N=69**  **n (%) or median (IQR)** | **Lower Probability^a^**  **N=114**  **n (%) or median (IQR)** | **P-value^b^** |
| --- | --- | --- | --- |
| **Clinical Probability Score Components** | | | |
| CXR findings score (0-4) |  |  | <0.001 |
| Normal (0) | 1 (1.5) | 41 (36.0) |  |
| Moderate (1) | 18 (26.1) | 72 (63.2) |  |
| Extensive(2+) | 50 (72.5) | 1 (0.9) |  |
| Constitutional symptoms ≥2 | 23 (33.3) | 16 (14.0) | 0.008 |
| TB contact history | 33 (47.8) | 43 (37.7) | 0.18 |
| Failure to thrive | 40 (58.0) | 36 (31.6) | <.001 |
| **CRP Performance** | | | |
| CRP ≥5 mg/L | 29 (42.0) | 36 (31.6) | 0.15 |
| CRP ≥10 mg/L | 25 (36.2) | 27 (23.7) | 0.07 |
| **Treatment Response (n=31 vs. 55)^¥^** | | |  |
| Median baseline CRP (IQR) | 2.5 (0.8, 19.3) | 1.8 (0.7, 11.6) | 0.24 |
| Near end treatment CRP | 0.6 (0.3, 1.7) | 0.8 (0.5, 2.5) | 0.21 |
| Within group change (p-value) | 0.001 | 0.008 |  |
| **Abbreviations:** CRP, C-reactive protein; CXR, chest X-ray; IQR, interquartile range; TB, tuberculosis.  Clinical Probability Scoring System: CXR findings: Normal=0, minimal=1, extensive=2;  Constitutional symptoms: <2 symptoms=0, 2-3 symptoms=1, >3 symptoms=2; TB contact: No contact=0, household contact=1; Growth parameters: Normal growth=0, failure to thrive=1.  ^a^ Higher probability defined as scoring ≥4 points; Lower probability defined as scoring <4 points.  ^b^ Values presented as median (IQR) for continuous variables and n (%) for categorical variables.  **^¥^** Treatment response analysis includes only participants who completed ≥4 months of treatment (n=31 for higher probability, n=55 for lower probability) | | | |
